# How Do Time Use and Social Relationships Affect the Life Satisfaction Trajectory of Korean Adolescents?

**DOI:** 10.3390/ijerph17051532

**Published:** 2020-02-27

**Authors:** Hyemee Kim, Heyjin Moon, Joan P. Yoo, Eunji Nam

**Affiliations:** 1 Department of Social Welfare, Institute of Social Science Research, Incheon National University, 119 Academy Road, Yeonsu-Gu, Incheon 22012, Korea; hkim@inu.ac.kr; 2 Seoul Welfare Foundation, 21 Baekbeom-ro 31-gil, Mapo-gu, Seoul 04147, Korea; 3 Department of Social Welfare, Seoul National University, 1 Gwanak-ro, Gwan-ak Gu, Seoul 08226, Korea; joanyoo@snu.ac.kr; 4 School of Social Work, College of Health Professions and Sciences, University of Central Florida, 12805 Pegasus Drive, Orlando, FL 32816, USA; enam@ucf.edu

**Keywords:** Life satisfaction trajectory, Korean adolescents, time use, social relationships, Korean Children and Youth Panel Survey

## Abstract

This study investigated the life satisfaction trajectory of Korean adolescents, and factors associated with changes in life satisfaction. Specifically, we focused on how changes in time use and social relationships were associated with changes in life satisfaction. Using three waves of the Korean Children and Youth Panel Survey, we conducted a series of multilevel growth curve modeling analyses. The results indicate that Korean adolescents’ life satisfaction decreased over a three-year period, and that time spent on leisure and sleeping were both significant predictors of changes in life satisfaction. Life satisfaction decreased at a slower rate for adolescents whose relationships with peers and teachers positively increased over time. Findings highlight the importance of ensuring adequate amount of sleep and providing various opportunities for leisure activities in improving Korean adolescents’ life satisfaction. Furthermore, social relationships, specifically with teachers and peers should be the focus of prevention and intervention for adolescents to maintain and improve their level of life satisfaction.

## 1. Introduction

Life satisfaction (LS), defined as subjective appraisal of quality of one’s overall life [1]. It is a measure often used to represent individuals’ overall well-being [1,2] and is considered as an important component of one’s mental health [3]. Importance of understanding LS in adolescents is particularly vital as LS of adolescent is associated with social, physical, and psychological functioning in adulthood [4]. Scholars argue that low LS during childhood and adolescence is associated with poor mental, physical, and social health. Particularly, those with lower LS are more likely to exhibit a greater number of depressive symptoms, undergo difficulties in interpersonal relations, and develop unhealthy lifestyles [3,5,6,7,8]. LS, being an important protective asset for adolescents’ healthy development [9], improvement of LS has been an important component of health promotion in many societies [10].

Understanding LS among adolescents is particularly important in Korea where adolescents’ LS level is known to be lower than that of adolescents in other societies. According to a recent report [11], Korean adolescents had the lowest overall LS compared to their counterparts in other countries. Moreover, high suicide rates among adolescents in Korea may be an alarming indication of their low LS. In sum, improving LS of adolescents and identifying early markers, risk and protective factors of LS remain as crucial tasks for public health related scholars, policy makers, and practitioners as well as the overall Korean society since the future generation is deemed to be at risk [9,11].

In response to the bleak present of Korean adolescents, a number of studies have examined the contributing factors of LS [12,13,14,15] as well as changes in LS [13,14,15]. Studies have commonly reported that adolescents’ self-esteem [13,15], social relationships [12,13,14,15], and academic achievement [14,16] are important determinants of their LS, and argued for a need to develop various measures to improve their self-esteem and strengthen their relationships with parents, peers, and teachers. Such finding is not limited to Korean studies as studies examining LS of adolescents in other societies have also reported significant relationships between LS and adolescents’ various psychological [8] and social functions [8,16,17,18]. Despite the contributions of earlier work on adolescents’ LS in shedding light on the nature of LS and its determinants, how LS changes over time remains unclear. LS, considering its dynamic, volatile nature, is known to change with time and events [14,17]. The need for longitudinal analyses to understand LS is recognized, and recent studies have expanded the understanding of LS by examining the changes in LS as well as factors associated with the changes [9,13,14,15,16,17,18,19,20,21].

In general, LS is known to decrease during adolescence [8,21,22]. However, the studies have been conducted heavily in western countries, and little is known about LS changes beyond non-Asian populations. In addition, findings pertaining to LS of Korean adolescents also remain inconclusive with mixed findings [13,14,15]. Furthermore, despite accumulated knowledge we have of potential determinants of adolescents’ LS, one area that has not yet received much attention is how adolescents spend their time and how it is related to their LS. Despite numerous studies investigating the relation between time use and LS in other age groups, such as the elderly [23], little is known about how adolescents’ time use is related to their LS. According to OECD [11], adolescents’ LS measures satisfaction with various aspects of their life, including peer relationships, feelings of the self, quality of family and community life, as well as their use of time. Yet, to this date, studies investigating adolescents’ time use in relation to their LS is overall limited. Even these handful of studies have only examined the association between LS and a particular domain of time use such as sleep [24,25], leisure [26,27], and studying [22]. For example, De Matos et al. [24] found that adolescents who sleep more than eight hours a day are more likely to report higher LS, while Park and Huebner [22] explained that adolescents who spend more time studying reported higher LS than their counterparts. Leisure time is also reported to be associated with adolescents’ LS, though the findings are inconsistent. Despite this rising concern on how adolescents spend their time and its association with LS, little is known about the changes in time use of adolescents and how such change in time use may be associated with their changing LS.

Social relationships are a vital part of adolescents’ lives [3,12]. In particular, relationships adolescents share with their peers and teachers are significantly influential on their overall well-being since much of their lives are spent in schools with teachers and peers [3,12,22]. Literatures have well documented the significance of teacher and peer relationship in relation to LS [3,12,13,14,15,28]. However, what has been overlooked in many of previous studies is the dynamic nature of these relationships. Adolescents’ perception and experiences of relationships with others, as well as LS, are also likely to change with time. In examining adolescents’ LS, it is crucial to account for such changes, and identify how changes in adolescents’ perception of their social relationships also affect their LS over time. However, studies to this date have not adequately addressed the long-term dynamic relations between social relationships and LS.

The present study aims to fill the gap in LS literature as it examines the change in LS among adolescents in Korea and how time use and social relationships may be associated with the changing LS along with other time-invariants variables. In doing so, the study seeks to expand the current understanding on adolescents’ LS and provide both policy and practice implications for development of various measures to improve adolescents’ overall LS.

## 2. Materials and Methods

### 2.1. Sample

To examine changes in LS of Korean adolescents and associated factors, we utilized waves 3, 4, 5 (2012–2014) of the middle school cohort from the Korean Children and Youth Panel Survey (KCYPS). The KCYPS is a nationally representative panel study conducted on children and youth in Korea every year and seeks to examine the causal links between the environment and the development of children and adolescents. In 2010, 2351 students who were in the 1st grade of 78 middle schools (equivalent of 7th grade in the U. S. school system) and their parent participated in the survey. The questionnaires were administered by computer-assisted self-interviews [29]. From 2010 to 2016, the data were collected every year, resulting in seven waves of data collection. The retention rate was 80.8% (N = 1881). We selected three waves (3–5) for analyses because key variables, such as LS, are only available in these three waves of data. We also did not include wave 6 in this study as data from wave 6 were collected after the college entrance exam, which may obscure the results of how adolescents spend time on a daily basis, and LS level may be particularly different after the college entrance exam [14,15].

We excluded 252 students whose parents chose not to participate. Critical information such as family structure and household income were collected in the parent survey. The total number of the sample included in wave 3 was 2191. The number of adolescents participating in wave 4 was 2062 and 1983 in wave 5.

### 2.2. Measures

The KCYPS measures LS using three items from the Life Satisfaction Survey by Diener et al. [1]: “I enjoy my life”, “I do not have much to worry about”, and “I am happy with my life”. Students were asked to choose their response on a 4-point Likert scale ranging from “strongly disagree”(1) to “strongly agree”(4). The Cronbach’s alpha ranged from 0.78 to 0.81 in three waves of data. We used the total score of these three items in each wave.

As for time-varying independent variables, we focused on adolescents’ time use and qualities of social relationships. Time use indicates the amount of time students spend on sleeping, studying, and leisure activities on a day. Study time includes time spent in private institutions for academic subjects after school, time spent on doing homework, and studying. Leisure time comprises of time spent on reading for pleasure, playing computer and electronic games, on Internet, watching television or movies, and hanging out with friends. Sleep time is the amount of time students spent on sleeping. The average amount of time per week was measured by summating the number of hours spent for each activity during the weekdays and weekends, respectively. For weekdays, the sum of hours was multiplied by five, and it was multiplied by two for weekends. The average amount of time spent on each activity per day was then calculated by totaling the number of hours and dividing the total number of hours by seven, resulting in the average time adolescents spent on sleeping, studying, and doing leisure activities on a daily basis. Information from all three waves of data was included in the analyses.

Another time-varying factor we included in the analytic model was adolescents’ perception of relationships they share with their peers and teachers. Peer and teacher relationships were measured using six items respectively. The measures are modified subscales of the School Adjustment Scale [30], including items such as “I get along with my classmates”, and “I tend to apologize first when I quarrel with a friend” for measuring peer relations, and “My teacher is nice to me”, “I feel comfortable talking to my teacher” for measuring teacher relationship. Adolescents were asked to rate how strongly they agree with the statements on a four-point Likert scale ranging from “I strongly agree” to “I strongly disagree”. The responses were reverse coded, and the higher score is indicative of stronger peer and teacher relationships.

Students’ gender and parents’ educational background were included as fixed socio-economic variables in this study. We assigned zero to male students and a score of one to female students. Parents’ educational background was also included as a binary variable with those with two years of college education and more were coded one, while those with less than two years of college education were coded zero.

### 2.3. Statistical Analysis

To identify factors associated with changes in LS among middle school adolescents, we employed multilevel growth curve modeling. Multilevel growth curve modeling is commonly used to analyze panel data, which contains repeated measurements of the same variables from the same individuals [31]. A major strength of multilevel growth curve modeling is that it allows tests of between-individual and within-individual variances simultaneously. By doing so, we are able to examine how time invariant and time varying variables could explain these two different levels of variances, respectively. Multilevel growth curve modeling estimates the growth function of the dependent variable at the level-1 (Equation (1)). The equations at level 2 estimates the factors that could explain the variances found in the intercept and the slopes of the time variables. Equation (1) shows the unconditional model of the multilevel quadratic growth curve model. The Equation (1): Multilevel growth curve model.

(1) $$\mathrm{Level}-1:\;Y_{ti}=\pi_{0i}+\pi_{1i}Time_{ti}+\pi_{2i}Time^{2}{}_{ti}\;+\;e_{ti}$$

$$\mathrm{Level}-2:\;\pi_{0i}=\beta_{\mathrm{oo}}+{\;\gamma }_{\mathrm{oi}}$$

$$\pi_{1i}={\;\beta }_{10}+{\;\gamma }_{1i}$$

$$\pi_{2i}={\;\beta }_{20}+{\;\gamma }_{2i}$$

In the level-1 equation (Equation (1)), *Y_ti_* is the measurement of the dependent variable for person *i* at time *t*. Time indicates the measurement points. If we assume that the first measurement point to be 0, *π_0i_* is the initial status of the person *i.* Lastly, *e_ti_* is the error term for person *i* at time *t*. It is assumed to have a normal distribution with a mean of 0 and common variance of *σ^2^* [31]. In the level-2 equation, $\beta_{00}$ represents the mean intercept, and $\beta_{10}$ represents the mean rate of change. There are two random effects γ_0*i*_ and γ_1*i*_, and are assumed to have variances of τ_00_ and τ_11_. Statistical significance of the variances indicates that there are enough differences across individuals to include independent variables to explain these variances, allowing researchers to fit the conditional models. Three different conditional models were estimated in this study. First, we included time invariant variables, gender and parent’s level of education to level-2 equation to examine how these variances explain between-individual differences. Equation (2). Conditional model with time-invariant variables in Level-2.

(2) $$\pi_{0i}={\;\beta }_{00}+\;\overset{k}{\underset{q=1}{\sum }}\beta_{0q}x_{qi}+{\;\gamma }_{0i}$$

$$\pi_{1i}={\;\beta }_{10}+\;\overset{k}{\underset{q=1}{\sum }}\beta_{1q}x_{qi}+{\;\gamma }_{1i}$$

Second, we estimated a conditional model with only time-varying variables as *Z_ti_* (e.g., time use and relationship variables). We assumed the time-varying variables *Z_ti_* have fixed effects on adolescents’ LS since we are interested in how changes in *Z_ti_* within individuals affect changes in the dependent variable, LS in this study. However, the effect of time-varying variables includes both within-individual differences across time and the mean differences between individuals [32]. In order to estimate the effect of within-individual differences across different time points on the dependent variable, we needed to include time-varying variable *Z_ti_* as a group-mean centered variable in level 1. We also included the individual mean of the time-varying variable $\bar{{Ζ}_{i}}$ in the level 2 (see equation 3) to account for overall individual differences. In this model, $\beta_{30}$ indicates the estimated effect of within-individual changes over time on the dependent variable, and $\beta_{0\rho }$ indicates the effect of the mean differences between individuals on the dependent variable [32]. Third, we estimated a combined model that includes both time-invariant and time-varying variables to understand how effects of time-varying variables change controlling for the effect of the time-invariant individual characteristics. For analyses, we used HLM(7.03) programs. Equation (3). Conditional model with only time-varying variables.

(3) $$\mathrm{Level}-1:\;Y_{ti}=\pi_{0i}+\pi_{1i}Time_{ti}+\pi_{2i}Time^{2}{}_{ti}\;+\;\pi_{3i}\left(Z_{ti}\right)+e_{ti}$$

$$\mathrm{Level}-2:\;\pi_{0i}=\beta_{00}+\;\overset{k}{\underset{\rho =1}{\sum }}\beta_{0\rho }{\bar{Z}}_{i}+\;\gamma_{0i}$$

$$\pi_{1i}=\beta_{10}+\;\overset{k}{\underset{\rho =1}{\sum }}\beta_{1\rho }{\bar{Z}}_{i}+\;\gamma_{1i}$$

$$\pi_{2i}=\beta_{20}+\gamma_{2i}$$

$$\pi_{3i}={\;\beta }_{30}$$

## 3. Results

### 3.1. Descriptive Anlaysis

#### 3.1.1. Individual and Family Characteristics

The total number of adolescents included in this analysis was 2191. The average number of participation across three waves of data collection is 2.8. The descriptive statistics for time-invariant individual and family characteristics are presented in Table 1. Among the participants, males consisted approximately 51% of the sample. Approximately 54% of parents had more than high school education.

#### 3.1.2. Changes in LS, Time Use, and Relationship Characteristics

Means and standard deviations of two time-varying independent variables, relationship and time use, along with the dependent variable, LS, are illustrated in Table 2. Korean adolescents’’ LS was found to show a slight decreasing trend from Year 1 to Year 3 from 2.85 (SD = 0.68) in Year 1, 2.83 (0.61) in Year 2, to 2.80 (SD = 0.59) in Year 3. The average number of hours adolescents spent sleeping were 7.86 h in Year 1, followed by 6.94 h in Year 2, and 6.75 h in Year 3, also showing a decreasing trend. Similarly, the average number of hours spent in leisure activities were 5.21 h in Year 1, and dropped significantly in Year 2 and 3 with 3.91 h 3.63 respectively. The average number of hours Korean adolescent spent in studying was 3.24 h per day which slightly dropped in Year 2 with 2.99 h, and bounced back to 3.15 h per day in Year 3. Peer relationship increased over the three year period with scores of 3.07 (SD = 0.39) in Year 1, 3.11 (SD = 0.38) in Year 2, and 3.15 (SD = 0.36) in Year 3. Teacher relationship, on the other hand, did not show much change with scores of 2.89 (SD = 0.63) in Year 1, 2.88 (SD = 0.58) in Year 2, and 2.90 (SD = 0.56) in Year 3.

### 3.2. HLM Growth Curve Analysis

#### 3.2.1. Unconditional Growth Model

The unconditional growth curve model was first fitted to examine the trajectory of change in LS among Korean adolescents. The unconditional model provides the average level of LS for Korean adolescents at Year 1, the average rate of change over a three year time period, and the amount of individual variability in these estimates. To find the best fitting model, both (a) linear, and (b) quadratic model were estimated. As shown in Table 3, results suggest that a quadratic growth model better fits the data than the linear growth model. The model comparison test yielded χ^2^ =33.02 (df = 3) significant at *p* < 0.001. The intercept of 2.842 indicates the mean score of LS at Year 1, while the slope of −0.010 (=−0.011+0.001) shows that their LS had a negative nonlinear trajectory. The results of the random effects indicate significant heterogeneity in coefficients of the intercept and the slope, suggesting the need for further examination on the inter-individual differences.

#### 3.2.2. Conditional Growth Model

To examine the effects of individual and family characteristics on the growth curve of LS, we included adolescents’ gender and parents’ education level at Year 1 as level 2 independent variables as shown in Table 4 (Model 1). Gender (β = −0.132, *p* < 0.001) was significantly associated with the LS intercept, indicating that male students reported a higher level of LS at Year 1. Parents’ education was significantly associated with the intercept (β = 0.071, *p* < 0.01) and the slope of the LS trajectory (β = −0.030, *p* < 0.05). Next, we examined the effect of two time-varying variables, time use, and relationship quality on LS trajectory (Model 2 and 3). Time use and relationship quality were entered to the models separately to first examine their unique effects. As shown in model 2 in Table 4, the amount of time used for sleeping, leisure, and studying were included as level 1 variables. The findings show that the mean amount of time adolescents spent on sleeping (β = 0.0008, *p* < 0.01) and studying (β = 0.0005, *p* < 0.001) were positively associated with their LS at Time 1 (intercept), indicating that those who spent more time in sleeping and studying, on average, had higher LS at Time 1. However, the change in LS (slope) was only predicted by the increase in leisure time (β = 0.004, *p* < 0.10), indicating that the LS decreased at a slower rate for those whose leisure time increased over time on average. Within-individual differences were also observed with sleeping time. The LS decreased more gradually for adolescents’ whose sleeping time increased over time (β = 0.590, *p* < 0.05).

The unique effects of relationship were also examined (Model 3). The results show that the adolescents’ relationship with peers (β = 0.485, *p* < 0.001) and teachers (β = 0.264, *p* < 0.001) were significantly associated with their initial LS, such that those who had stronger relationships with peers and teachers reported higher LS at Time 1. Results also show that within-individuals change in LS was significantly associated with teacher (β = 0.104, *p* < 0.001) and peer (β = 0.213, *p* < 0.001) relationships, indicating that adolescents whose relationships with teachers and peers became stronger over the three-year period had their LS decrease at a slower rate.

The combined model was then estimated to examine the effects of both time-variant and time-invariant variables simultaneously (Model 4). The effect of gender remains significant at *p* < 0.001 level with male adolescents reporting higher LS at Time 1. Parents’ education background (β = 0.057, *p* < 0.05) was also significantly associated with LS at Time 1. The sleeping time (β = 0.021, *p* < 0.001), peer relationship (β = 0.534, *p* < 0.001) and teacher relationship (β = 0.240, *p* < 0.001) were associated with the LS at Time 1. Results also revealed the effect of within-individual changes on LS trajectory was associated with changes in sleep time and relationships. Adolescents whose sleeping time increased over a three-year period and whose relationships with peers and teachers became stronger had their LS decrease at a slower rate than the counterparts. Lastly, the final model was estimated, including the variables that reached statistical significance in the earlier models. The findings show that those who spent more time on sleeping on average, and those who had, on average, stronger peer and teacher relationships also had higher LS at Time 1. In terms of changes in LS, inter-individual differences were observed with leisure time (β = 0.004, *p* < 0.01), indicating that the adolescents who, on average, spent more time in leisure activities over a three-year period had their LS decrease at a slower rate. On the other hand, changes in sleep time (β = 0.657, *p* < 0.001) were significantly associated with within-individual changes in LS. Changes in peer (β = 0.213, *p* < 0.001) and teacher (β = 0.101, *p* < 0.001) relationships were also associated with the within-individual changes in LS. In other words, LS decreased at a slower rate for adolescents whose sleep time increased, and relationships with peers and teachers grew stronger over a three year span.

## 4. Discussion

LS, an important indicator of one’s overall well-being [1,2], is an important topic to investigate in Korea where adolescents’ LS is known to be relatively low and youth suicide rate is on a continuous rise [11]. This study examined how LS changes over time and identified factors associated with changes in LS, specifically focusing on time use and social relationships. In doing so, we analyzed time use and social relationship in forms of both time invariant and time-variant variables. This approach allowed us to understand how changes in time use and social relationships among adolescents explain changes in LS over time. The findings are summarized as follows.

First, in the current study, an overall trajectory of LS decreased over a three year period of time for Korean adolescents, consistent with previous studies [8,21,22]. However, the finding is different from that of previous studies on Korean adolescents. For example, Yoo et al. [15] reported an upward trajectory of LS in their study. However, this may be due in part to the differences in the ages of participants. In the current study, we excluded wave 6 to control the effect of college entrance exam whereas Yoo et al. [15] included the data up to a year after high school graduation. What is evident in the Korean literature is that adolescents’ stress and LS levels change significantly after the college entrance exam [13,15]. In general, many adolescents in Korea tend to experience stress related to their academic achievement to get into good colleges, which may cause low LS during high school years [15]. Taken together, it appears that adolescents’ LS continuously decreases up to junior year in high school, although it could bounce back once they graduate from high school. Such finding provides important information on the main target of intervention and the time frame during which active interventions should be provided.

The results also revealed a significant gender difference in LS trajectory. Male adolescents had a higher LS level at Time 1 compared to female adolescents. However, the effect of gender was not significant in LS slope, indicating that the gap between the male and female adolescents’ LS remained through a three-year period. Such gender difference in LS is well-reported both in Korean studies and studies in other countries [11,12,13,14,15,21]. Our finding confirms and highlights the need to understand why female adolescents’ LS is lower and to focus on developing gender-specific interventions to promote female adolescents’ overall LS.

It was interesting to observe the effect of parents’ education background on LS. LS of adolescents whose parents had more than college education was higher than that of adolescents whose parents had comparatively less education. Also, a difference in LS levels due to parental education remained consistent over time. The finding is alarming as it suggests that adolescents’ LS may be determined by their parents’ education background. Education is one dimension of socioeconomic status [33]. It is concerning as it appears that even happiness among adolescents is impacted by parental factors. It is not yet clear why parents’ educational background is associated with adolescents’ LS. One explanation is that more educated parents are likely to earn higher income which can provide a better environment for their children [16]. In fact, previous studies have shown that household income is associated with academic achievement of their children in Korea [34]. Given the importance of academic achievement in understanding LS among adolescents, future study should further examine the mediating or moderating role of parents’ education in LS.

An important finding of this study is that changes in time use of adolescents are associated with the LS trajectory. Sleeping time was an important determinant for Korean adolescents’ LS in the current study. LS was higher among adolescents who slept more, and such difference remained consistent over time. In addition, LS decreased at a slower rate for adolescents whose sleeping time increased over time. This suggests the importance of sleeping time for adolescents’ healthy development [24]. The finding is particularly pertinent in Korean society where adolescents’ sleep time on average is notably less than the eight to ten hours recommended by the National Sleep Foundation in the U.S. for adolescents between the ages of 14 and 17 years [35]. A recent national statistics reported that Korean adolescents between the ages of 13 and 18 years slept 7 h and 28 min on average in 2017 [36], and sleeping time decreased with their ages as shown in this study. It is also reported that a large portion of Korean adolescents feel that their sleeping time is inadequate [37]. Instead, they spend much of their time at school or at the after-school academic institutes, and spend the vast majority of their time on studying and doing homework [37]. We found that increasing adolescents’ sleeping time is associated with a slower decrease in LS, providing an important insight into adolescents’ well-being. As their LS is closely related to how much they sleep, ensuring adolescents get an adequate amount of good quality sleep can be a point of intervention in improving their overall well-being, including LS. Sleep is a critical part of life, providing restoration and renewal of energy [24]. The findings suggest that social workers and public health care providers working with adolescents must address sleep deprivation among adolescents to improve their overall well-being.

Another important aspect of daily time use in relation to adolescents’ LS was leisure time. In the current study, leisure time was defined as the time adolescents used for reading, watching television and/or movies, spending time with friends, and playing computer (on-line games and/or other computer related activities). The finding shows that adolescents who on average spent more time in leisure activities had their LS decrease at a slower rate, indicating that adolescents’ leisure time is another important factor having a positive long-term effect on their LS. Along with sleeping time, the amount of time Korean adolescents spend on doing leisure activities also decreased over a three year period as shown in this study. A recent national statistic also reports a similar finding that the amount of time adolescents spend in leisure activities is inversely proportional to their age: The older adolescents spend the least amount of time in leisure activities [36]. Nearly 50 percent of adolescents spend less than two hours per day in leisure activities despite the fact that participation in leisure activities is an important source for adolescents’ healthy development providing various social, physical, emotional, and psychological benefits [25,26]. As the finding indicates, securing leisure time in adolescents’ daily lives is critical in improving their LS. Parents, schools, public health actors, and social workers need to acknowledge the importance of the leisure time in adolescents’ overall well-being. School curriculum needs to be revised to ensure the time for adolescents to engage in various leisure activities of their choice. Parents and local communities need to work together to provide more opportunities to adolescents to engage in healthy leisure activities.

On the other hand, the amount of time Korean adolescents spend in studying was not significantly associated with changes in LS. A recent OECD report [11] indicated that 23 percent of 15-year-old Korean adolescents spend more than 60 h per week in studying both in and out of school. Given that Korean adolescents spend an average of 10 h at schools on a daily basis, it is likely that Korean adolescents spend more than 12 h studying per day. However, as shown in this study, the number of hours spent in studying is not necessarily related to adolescents’ LS. Study time does not make any differences above and beyond sleep time and leisure time do in terms of LS. This may be a unique characteristic of Korean society where high academic achievement and long hours of studying are regarded as desirable, and it is possible that adolescents accept this cultural context naturally. Thus, the impact of study time may be minimal in affecting their overall LS.

Consistent with previous studies [3,12,13,14,15,28], adolescents’ social relationships also play a significant role in determining their LS over time. Adolescents’ perceived relationships with peers and teachers were significantly associated with their LS at Time 1 as well as changes in LS over a three-year period. In addition, positive changes in social relationships predicted slower decline in LS trajectory, indicating both peer and teacher relationships function as important protective factors for adolescents’ long-term LS. An ample amount of literature on children and adolescents has highlighted the importance of social relationships [13,14,15,22,28,38]. In particular, peer relationships are critical as they have both direct and indirect impact on adolescents’ well-being. Specifically, positive social relationships predict higher LS for adolescents [13,14,15]. They are also an important source of social support, buffering the impact of negative and stressful life events on their well-being [3]. What is relatively unknown is how relationships with teachers influence adolescents’ LS and well-being. Teachers may not be the source of direct emotional support like peers. John-Akinola [38] saw teachers as an environmental factor that influences adolescents’ well-being. As many Korean adolescents spend on average, more than 10 h a day at school [36], their school life becomes more meaningful and adolescents’ relationships with teachers may take up an important portion of such school life.

Adolescence is a period in life that exhibits a great degree of heterogeneity. LS among adolescents also can be very different between individuals. According to Salmela-Aro [20], Finnish adolescents have three trajectories of LS while they transit into post-compulsory education: high-decreasing, low-increasing, and high-stable. In the current study, a large amount of variances remained unexplained even in the final model that includes known predictors of LS. Future studies should explore the heterogeneity within LS among Korean adolescents as well as the mechanism explaining such heterogeneity. Future studies should also account for other important time-variants in examining LS trajectory of adolescents. Due to limitations of using the secondary data, we could not include some of the critical factors known to be associated with LS such as self-esteem [13,15], adolescents’ external and/or internal behavior [15], and relationship with parents [14,15]. A more comprehensive model accounting the effect of such time-varying factors would expand our understanding of LS further. Lastly, measurement limitation should also be acknowledged. As we used the secondary data which measured LS using only three items, future studies should employ a more comprehensive tool in measuring LS from various aspects to better capture the concept of LS.

## 5. Conclusions

This study investigated the trajectory of LS among adolescents in Korea. Over three years, LS among participants showed a decreasing trend. The most important findings in this study are the role of time use and social relationships in explaining LS change among adolescents in Korea. Among sleep time, study time, and leisure time, sleep time appears to be the most important type of time use in relation to LS. Relationships with peers and teachers appear to be equally important to LS among Korean adolescents. The findings highlight the importance of accounting for adolescents’ time use in understanding their LS, and how their LS is heavily impacted by the relationships they share with teachers and peers. To improve the LS of Korean adolescents, public health actors, educators, social workers, and parents need to be more assertive in ensuring adequate amount of sleep and leisure time for these adolescents, and specifically focus on assisting them to build healthy relationships with their peers and teachers.

## Figures and Tables

**Table 1:** Descriptive statistics of Korean adolescents.

Variables	Category	N (%)
Gender	Male	1113 (50.8)
	Female	1080 (49.3)
Parent’s Education	High school graduate or less	1005 (45.9)
	More than high school.	1185 (54.1)

**Table 2:** Changes in key variables.

Variables	Year 1 Mean (SD)	Year 2 Mean (SD)	Year 3 Mean (SD)
Life satisfaction (LS)	2.89 (0.63)	2.88 (0.58)	2.90 (0.56)
Average hours of sleep/day	2.85 (0.68)	2.83 (0.61)	2.80 (0.59)
Average hours of leisure activities/day	7.86 (0.59)	6.94 (1.00)	6.75 (1.03)
Average hours of studying/day	5.21 (2.32)	3.91 (2.18)	3.63 (2.22)
Peer relationship	3.24 (2.18)	2.99 (2.25)	3.18 (2.39)
Teacher relationship	3.07 (0.39)	3.11 (0.38)	3.15 (0.36)

**Table 3:** Unconditional growth model of LS.

Effect	Category	Linear Model	Quadratic Model
		Coefficient SE	Coefficient SE
Fixed effect	Intercept	2.846 *** 0.014	2.842 *** 0.015
	Linear slope	−0.020 ** 0.008	0.001 0.026
	Quadratic slope		−0.011 0.012
Random effect	Intercept	0.253 ***	0.296 ***
	Linear slope	0.02	0.299 ***
	Quadratic slope		0.039 ***
	Level-I error	0.187	0.16

** *p* < 0.01, *** *p* < 0.001.

**Table 4:** Conditional growth model of LS.

Effect	Time-invariant Variables Model (Model 1)		Time-varying Variables Model				Combined Model (Model 4)		Final Model	
			Time Use		Relationship					
			(Model 2)		(Model 3)					
	Coefficient	SE	Coefficient	SE	Coefficient	SE	Coefficient	SE	Coefficient	SE
**Fixed effects**										
**For Intercept**										
Intercept	2.869 ***	0.025	2.824 ***	0.016	2.852 ***	0.014	2.871 ***	0.026	2.868 ***	0.023
Gender	−0.132 ***	0.028					−0.139 ***	0.026	−0.124 ***	0.02
Parent’s ed.	0.071 **	0.028					0.057 *	0.027	0.051 +	0.027
sleep t.			0.0008 **	0.0003			0.021 **	0.007	0.022 ***	0.005
leisure t.			0.0002	0.0001			0.004	0.003		
study t.			0.0005 ***	0.0001			0.001	0.008	0.000	0.002
peer rel.					0.485 ***	0.052	0.534 ***	0.053	0.525 ***	0.04
teacher					0.264 ***	0.034	0.240 ***	0.034	0.234 ***	0.027
**For Linear slope**										
Intercept	0.015	0.029	0.042	0.03	−0.009	0.026	0.033	0.032	0.037	0.029
Gender	0.004	0.015					0.015	0.015		
Parent’s ed.	−0.030 *	0.015					−0.02	0.016	−0.017	0.015
sleep t.			0.001	0.004			0	0.004		
leisure t.			0.004+	0.002			0.003	0.002	0.004 **	0.001
study t.			0	0.002			0.001	0.002		
peer rel.					−0.014	0.03	−0.006	0.031		
teacher					−0.006	0.02	−0.007	0.02		
Sleep time			0.590 *	0.237			0.604 **	0.233	0.657 **	0.229
Leisure time			0.114	0.109			0.096	0.107		
Study time			−0.107	0.118			−0.166	0.116		
Peer rel.					0.213 ***		0.215 ***	0.027	0.213 ***	0.027
Teacher rel.					0.104 ***		0.103 ***	0.016	0.101 ***	0.016
**For Quadratic slope**										
Intercept	−0.01	0.012	−0.021 +	0.013	−0.011	0.012	−0.02	0.013	−0.020 +	0.012
**Random effects**										
Intercept	0.292 ***		0.293 ***		0.251 ***		0.242 ***		0.243 ***	
Linear Slope	0.302 ***		0.307 ***		0.293 ***		0.289 ***		0.295 ***	
Quadratic slope	0.040 ***		0.041 ***		0.040 ***		0.038 ***		0.040 ***	
Level-1 error	0.16		0.159		0.154		0.155		0.154	

+ *p* < 0.1, * *p* < 0.05, ** *p*< 0.01, *** *p* < 0.001.
